# Metabolic engineering of plant secondary metabolites: prospects and its technological challenges

**DOI:** 10.3389/fpls.2023.1171154

**Published:** 2023-05-12

**Authors:** Asem Mipeshwaree Devi, Khomdram Khedashwori Devi, Pukhrambam Premi Devi, Moirangthem Lakshmipriyari Devi, Sudripta Das

**Affiliations:** Plant Bioresources Division, Institute of Bioresources and Sustainable Development, Imphal, Manipur, India

**Keywords:** CRISPR/Cas9, secondary metabolism, flavonoids, metabolite engineering, prime editing guide RNA

## Abstract

Plants produce a wide range of secondary metabolites that play vital roles for their primary functions such as growth, defence, adaptations or reproduction. Some of the plant secondary metabolites are beneficial to mankind as nutraceuticals and pharmaceuticals. Metabolic pathways and their regulatory mechanism are crucial for targeting metabolite engineering. The clustered regularly interspaced short palindromic repeats (CRISPR)/Cas9-mediated system has been widely applied in genome editing with high accuracy, efficiency, and multiplex targeting ability. Besides its vast application in genetic improvement, the technique also facilitates a comprehensive profiling approach to functional genomics related to gene discovery involved in various plant secondary metabolic pathways. Despite these wide applications, several challenges limit CRISPR/Cas system applicability in genome editing in plants. This review highlights updated applications of CRISPR/Cas system-mediated metabolic engineering of plants and its challenges.

## Introduction

Plants produce a broad spectrum of secondary metabolites with diverse chemical properties. These metabolites are a means of communicating with nature, for their survival and an adaptation to the local environment (Erb and Kliebenstein, 2020). Because of their usefulness and productivity, plant-originated secondary metabolites have been part of human welfare. These metabolites are exploited for their nutritive values, utilized as a source of medicinal components, and agrochemicals. Our growing understanding of the biosynthetic pathways and identification of important targets make the engineering of secondary metabolism possible. For example, the synthesis of aromatic compounds in plants can be bioengineered using the shikimate pathway (Averesch and Krömer, 2018) and for terpenoids the isoprenoid pathway (Chatzivasileiou et al., 2019). Similarly, targeted engineering can be accomplished on specific functional groups that serve as an identifier for particular plant secondary products, e.g. monoterpenoids (essential oils), carotenoids (flower/fruits colours), or sesqui-, di- and triterpenes (phytoalexins, plant hormones).

For primary metabolism, regulation of the biosynthetic pathway occurs at the genetic level, but certain biosynthesis is stimulated by external factors like environmental stresses. These external factors induce a cascade of reactions by producing different signalling molecules (Ashraf et al., 2018), e.g. reactive oxygen species (ROS) trigger the biosynthesis of apocarotenoids (Hussain et al., 2018). However, in the case of secondary metabolism, regulation occurs mainly at the enzymic level or during subcellular compartmentation (e.g. isoquinoline alkaloid berberine biosynthesis). The targets for such regulations are usually the regulatory genes of the biosynthetic pathway or the transporter genes. Metabolic engineering for enhancing the secondary metabolite(s) biosynthesis either *in vitro* or by introducing a foreign gene has been reported using *Agrobacterium*-mediated transformation (Bomzan et al., 2022). The success efficacy of this method of gene modification was still low. Many a time, the intended mutation is accompanied by other non-specific mutations resulting in the accumulation of toxic intermediates.

With the development of genome sequencing technology, the evolution of genetic engineering in plants particularly in the area of the breeding program has entered a new phase. Molecular markers developed make it possible to generate comprehensive genetic and linkage maps to determine quantitative trait loci (QTLs) of agronomic importance. Once a gene of importance is identified, available genome editing tools can be implemented to provide a more specific gene modification. A few of the molecular tools that are more proficient in genome editing are homologous recombination, zinc finger nucleases (ZFNs), transcription activator-like effector nucleases (TALENs), tetratricopeptide repeat proteins, clustered regularly interspaced short palindromic repeats (CRISPR)-CRISPR associated protein 9 (Cas9), adenine base editor, ribonucleic acid interference (RNAi), site-directed sequence editing, oligonucleotide-directed mutagenesis, cisgenesis, intragenesis, and plastid genome and synthetic genomics. Among these tools, ZFNs, TALENs, and CRISPR/Cas systems are more frequently used for precise genome editing in plants. Metabolic engineering of the secondary metabolites with precise gene editing tools could overcome problems related to limited resources, availability, productivity, and diverse plant secondary metabolites. In this review, we provide an overview of the recent updates in the use of these technologies for engineering secondary metabolite production with main emphasis on the CRISPR-Cas system and its technological challenges.

## Developing trends in plant metabolic engineering

**Figure 1:**
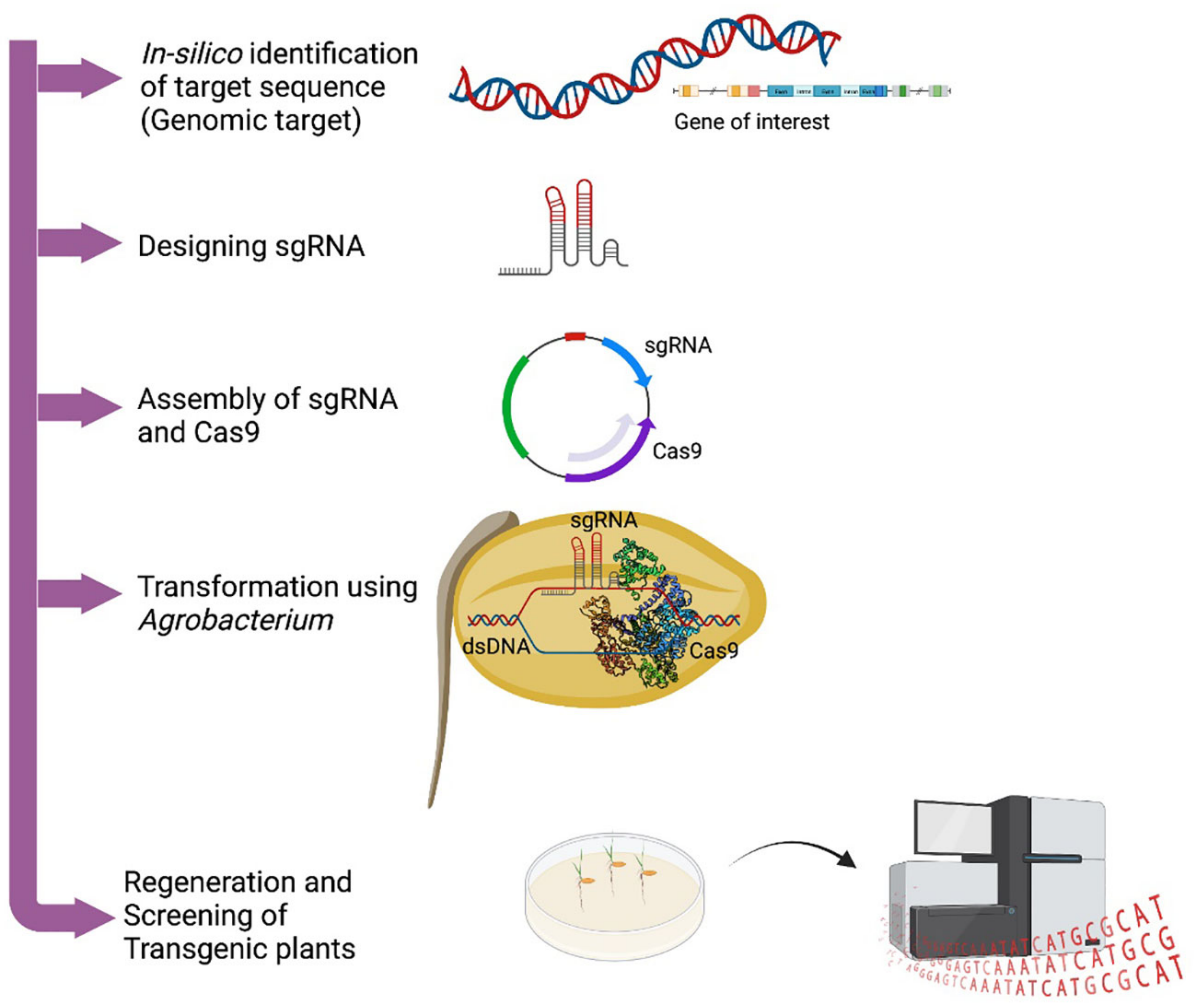
Gene manipulation by CRISPR-Cas gene editing system.

Genome editing using ZFNs was successfully reported in *Arabidopsis*, tobacco, maize, wheat, rice, and other plants for crop improvement related to yield, herbicides tolerance, etc. (Shukla et al., 2009; Li et al., 2012; Ran et al., 2018). However, not much work has been reported in metabolic engineering. Besides their role in targeting any DNA sequence in the genome, ZFNs have some limitations in high off-target bindings, and low affinity to AT-rich regions, and their construction is also challenging. TALENs have been used for precise genome editing in several plants including *Arabidopsis*, rice, barley, soybean, and maize to improve qualitative and functional genomics studies. Despite being a powerful gene editing technology, TALENS have limitations in its inability to edit methylated target sites, moreover delivery method was challenging due to its large size. A more widely accepted plant gene editing technique was the launch of the CRISPR/Cas system. Historically, CRISPR/Cas system was first recognized in bacteria as an adaptive immune system whereby the DNA of invading virus is degraded *via* RNA-guided DNA cleavage by Cas proteins (Horvath and Barrangou, 2010). Transcript of a short DNA sequence from a previous viral infection was integrated within the CRISPR locus to direct the recognition of the pathogenic DNA by the Cas nuclease (Jinek et al., 2012). Following the successive understandings of the binding motif and mechanism of Cas nucleases, the systems have been simplified for genome engineering in plants and other organisms (Jinek et al., 2012; Jiang et al., 2013) (**Figure 1**). **Table 1** lists a profile of the use of the CRISPR-Cas system for gene editing for production of plant secondary metabolites.

**Table 1:** Recent progress of genome editing for production of plant secondary metabolites using CRISPR/Cas9.

Plant species	Family	Target gene	Promoter for sgRNA	Secondary metabolites	Editing type	Method of delivery	Reference
*Arachis hypogaea* L.	Fabaceae	*ahFAD2*	MtU6	oleic acid	Targeted editing	*Agrobacterium rhizogenes*-based transformation	(Yuan et al., 2019).
*Atropa belladonna* L.	Solanaceae	*AbH6H*	U6-26	Hyoscyamine	Targeted mutagenesis	*A. tumefaciens* EHA105-mediated freeze thaw	(Zeng et al., 2021)
*Brasssica napus* L.	Brassicaceae	*BnaFAD2*	U3	Oleic acid, linolenic acid, linolenic acid	Knock out	*A. tumefaciens* mediated hypocotyl method	(Huang et al., 2020)
*Brasssica napus* L.	Brassicaceae	*BnaA.FAD2*	AtU6	Oleic acid	Knockout	*A. tumefaciens* GV3101 mediated	(Okuzaki et al., 2018)
*Camellia sinensis*	Theaceae	*CsHB1 yhNMT1*	At U3d	caffeine	mutated directionally.	*Agrobacterium tumefaciens*-mediated	(Ma et al., 2021)
*Camelina sativa* (L.) Crntz	Brassicaceae	*FAD2*	U3, U6	Oleic acid, PUFA	Targeted mutagenesis	*A. tumefaciens mediated* floral dip method	(Morineau et al., 2017)
*Camelina sativa* (L.) Crntz	Brassicaceae	*FAD2*	U6	Oleic acid, PUFA	Knockout	*A. tumefaciens* mediated floral dip method	(Jiang et al., 2017)
*Camelina sativa* (L.) Crntz	Brassicaceae	*FAE1*	U6- 26	Oleic acid or α-linolenic acid	Knockout	Floral vacuum infiltration method	(Ozseyhan et al., 2018)
*Camelina sativa*	Brassicaceae	*FAD2*	CsU3, CsU6	Oleic acid	Target mutagenesis	*Arabidopsis* floral-dip method	(Morineau et al., 2017)
*Camelina sativa*	Brassicaceae	*FAD2*	AtU6	Oleic acid	Knockout	Floral deep transformation	(Jiang et al., 2017).
*Camelina sativa*	Brassicaceae	*FAE1*	At U6-26	Oleic acid	Knockout	*Agrobacterium tumefaciens*-mediated	(Ozseyhan et al., 2018)
*Cannabis sativa* L	Cannabinaceae	*PDS*	AtU6	Phytocannabinoids	gene editing	*Agrobacterium*-mediated transformation	(Zhang et al., 2021)
*Cichorium intybus* var. *sativum*	Asteraceae	*CiGAS*	AtU6	sesquiterpene lactones	knockout	PEG transfection	(Cankar et al., 2021)
*Dendrobium officinale Kimura and Migo*	Orchidaceae	*C3H, C4H, 4CL, CCR,IRX*	OsU3	Alkaloids, phenanthrenes, polysaccgarides, bibenzyls essential oils, glycoside	Knockout	*A. tumefaciens* mediated	(Kui et al., 2017)
*Dioscorea alata L.*	Dioscoreaceae	*DrPDS*	DaU6	Carotenoid precursor	Knockout	*A. tumefaciens* mediated	(Syombua et al., 2021)
*Dioscorea zingiberensis C.H. Wright*	Dioscoreaceae	*Dzfps*	OsU3	Diosgenin	Targeted mutagenesis	*A. tumefaciens* GV3101 mediated	(Feng et al., 2018)
*Dioscorea zingiberensis*	Dioscoreaceae	*Dzfps*	OsU3	Squalene	Directed mutagenesis	*Agrobacterium tumeficians*-mediated transformation.	(Feng et al., 2018)
*Euphorbia pulcherrima*	Euphorbiaceae	*F3’H*	AtU6-26	Pelargonidin	Targeted mutagenesis	*Agrobacterium tumefaciens*	(Nitarska et al., 2021)
*Fagopyrum tataricum*	Polygonaceae	FtMYB45	AtU6-1	flavonoids	Knockout	*Agrobacterium rhizogenes*-mediated transformation.	(Wen et al., 2022)
*Glycine max (L.) Merr*	Fabaceae	*GmF3H1, GmF3H2* and *GmFNS-1*	GmU3, GmU6	Isoflavone	Directed mutagenesis	*Agrobacterium*-mediated cot-node transformation	(Zhang et al., 2020b)
*Glycine max*	Fabaceae	*GmFAD2-1A* *GmFAD2-1B*	AtU6	Oleic acid	Knock out	*Agrobacterium tumefaciens*-mediated	(Fu et al., 2022)
*Glycine max*	Fabaceae	*FAD2-1B* and *FAD2-1A* genes		oleic acid	Targeted mutagenesis	PEG-mediated transformation	(Kim et al., 2017)
*Glycine max*	Fabaceae	*GmUGT*	GmU6	Flavonoid	targeted mutagenesis	*Agrobacterium*-mediated transformation	(Zhang et al., 2022b)
*Glycine max (*L.) Merr.	Fabaceae	*IFS*	GmU3 or GmU6	Isoflavonoids	Targeted mutagenesis	*A. rhizobium* mediated hairy root culture	(Zhang et al., 2020b)
*Hordeum vulgare*	Poaceae	*HvHGGT*, *HvHPT*	OsU3	enhancing tocotrienols and tocopherol	knockout	*Agrobacterium tumefaciens*	(Zeng et al., 2020)
*Humulus lupulus* L.	Cannabinaceae	*PDS*	U6-626p and U6-29p	Carotenoids	Targeted mutagenesis	*A. tumefaciens mediated*	(Awasthi et al., 2021)
*Ipomoea nil*	Convolvulaceae	*lnCCD4*	AtU6	Carotenoid	Targeted mutagenesis	*Agrobacterium*-mediated transformation	(Watanabe et al., 2017)
*Ipomoea nil*	Convolvulaceae	*DFR-B*	AtU6	Carotenoid	Knock out	*Agrobacterium*-mediated transformation	(Watanabe et al., 2017)
*Ipomoea nil*	Convolvulaceae	*DFR-B*	*AtU6*	Anthocyanin	targeted mutagenesis	*Agrobacterium*-mediated transformation	(Watanabe et al., 2017)
*Medicago truncatula*	Fabaceae	*CYP93E2*	AtU6-26	Soyasapogenols	knockout	Agrobacterium tumefaciens	(Confalonieri et al., 2021)
*Musa* sp.	Musaceae	*LCYϵ*	OsU3	β-carotene	Targeted mutagenesis	*Agrobacterium* mediated genetic transformation	(Kaur et al., 2020)
*Nicotiana tabacum*	Solanaceae	*BBL*	AtU6-26	Nicotine	CRISPR/Cas9 knockout	*Agrobacterium*-mediated transformation	(Schachtsiek and Stehle, 2019)
*Nicotiana tabacum L.*	Solanaceae	*FucT, XylT*	U6	Alkaloids, flavonoids, Terpenoids, phenylpropanoids	Knockout	*A. tumefaciens* (EHA105, LBA4404) mediated	(Mercx et al., 2017)
*Nicotiana tabacum*	Solanaceae	*XylT*, *FucT*	U6	Glycans	CRISPR/dCas9 knockout	*Agrobacterium* mediated genetic transformation	(Mercx et al., 2017)
*Oryza sativa*	Poaceae	*Badh2*	OsU6	2-acetyl-1-pyrroline	Directed mutagenesis	–	(Shao et al., 2017)
*Oryza sativa*	Poaceae	*OsBADH2*	U6a	2-acetyl-1-pyrroline	Base editing	*Agrobacterium*-mediated transformation.	(Tang et al., 2021)
*Oryza sativa*	Poaceae	*OsBADH2*	OsU6	Aromatic compounds viz., pyrrolidine, pyridine, pyrazine, pyradazine and pyrozole	Directed mutagenesis	*Agrobacterium*-mediated transformation	(Ashok Kumar et al., 2020)
*Oryza sativa*	Poaceae	*OsBadh2*.	OsU6	2-acetyl-1- pyrroline (2-AP)	Directed mutagenesis	*Agrobacterium*-mediated transformation.	(Hui et al., 2022)
*Oryza sativa*	Poaceae	*Osor*	Osu6	β Carotene	genome editing	*Agrobacterium*	(Endo et al., 2019)
*Oryza sativa*	Poaceae	*CCD7*	OsU3	Strigolactones (SLs)	targeted mutagenesis	Agrobacterium-mediated transformation	(Butt et al., 2018)
*Oryza sativa*	Poaceae	*GR2* cassette insertion	Osu6	β Carotene	Gene insertion	Particle bombardment	(Dong et al., 2020)
*Papaver somniferum* L.	Papaveraceae	*4’OMT2*	AtU6p	Alkaloid	Knockout	*A. tumefaciens* mediated leaf infiltration	(Alagoz et al., 2016)
*Papaver somniferum*	Papaveraceae	*4’OMT*	AtU6	benzylisoquinoline alkaloids (BIAs) reduced	CRISPR/Cas9 knockout	*Agrobacterium*-mediated transformation	(Alagoz et al., 2016)
*Salvia miltiorrhizaBunge*	Lamiaceae	*SmRAS*	U3	Rosmarinic acid, phenolic acids, diterpenoids	Knockout	*A. rhizogenes* hairy root culture	(Zhou et al., 2018)
*Salvia miltiorrhiza*	Lamiaceae	*SmRAS*	AtU6	Rosmarinic acid and lithospermic acid B	Precise editing/specific mutation	*Agrobacterium rhizogenes* mediated transformation	(Zhou et al., 2018)
*Salvia miltiorrhiza*	Lamiaceae	*SmLAC*	AtU6	Salvianolic acid B	Knockout	*Agrobacterium rhizogenes* mediated	(Zhou et al., 2021)
*Salvia miltiorrhiza*	Lamiaceae	*SmCPS1*	AtU6	Tanshinones (diterpenes)	Knockout	*Agrobacterium rhizogenes*-mediated transformation	(Luo et al., 2014)
*Salvia miltiorrhiza*	Lamiaceae	*bZIP2*	AtU6	phenolic acid	Knockout	*Agrobacterium tumefaciens*	(Shi et al., 2021)
*Salvia miltiorrhiza*	Lamiaceae	*SmRAS50*	AtU3 (sgRNA) AtUBQ	phenolic acids (rosmarinic acid)	Knockout	*Agrobacterium rhizogenes*- mediated transformation	(Zhou et al., 2018)
*Salvia miltiorrhiza*	Lamiaceae	*SmCPS1*	AtU6	Tanshinones	CRISPR/dCas9 knockout	*Agrobacterium rhizogenes*- mediated transformation	(Mercx et al., 2017)
*Solanum lycopersicum* cv. Ailsa Craig (AC)	Solanaceae	*lncRNA1459*	35S	Ethylene, Lycopene downregulation	Knockout	*A. tumefaciens* mediated	(Li et al., 2018)
*Solanum lycopersicum*	Solanaceae	*CCD8*	AtU6-26	Carotenoid accumulation	Targeted mutagenesis	*Agrobacterium tumefaciens*	(Bari et al., 2019)
*Solanum lycopersicum*	Solanaceae	*SlS5αR2*	AtU6	α-tomatine level decreased	Knockout	*Agrobacterium rhizogenes*	(Akiyama et al., 2019)
*Solanum lycopersicum*	Solanaceae	*GABA-TP1, GABA-TP2, GABA-TP3, CAT9 and SSADH*	AtU3d, AtU3d, AtU3b, AtU3b, AtU6-1 or AtU6-29	γ-aminobutyric acid (GABA) enhanced	Precise editing	*Agrobacterium*-mediated transformation	(Li et al., 2018)
*Solanum lycopersicum*	Solanaceae	*Psy1, CrtR-b2*	AtU6	Carotenoid	Directed mutagenesis	*Agrobacterium*-mediated transformation,	(D’Ambrosio et al., 2018)
*Solanum tuberosum*	Solanaceae	*StSSR2*	AtU6	Steroidal glycoalkaloids	Knockout	*Agrobacterium* tumefaciens	(Zheng et al., 2021)
*Symphytum officinale*	Boraginaceae	*HSS*	At U6-26	pyrrolizidine alkaloids	Directed mutagenesis	*A. rhizogenes* mediated transformation	(Zakaria et al., 2021)
*Vitis vinifera*	Vitaceae	*VvBZIP36*	AtU6-1, -AtU6-29, AtU3d, and -AtU3b	Anthocyanin	Knockout	*Agrobacterium tumefaciens*	(Tu et al., 2022)
*Zea mays*	*Poaceae*	*ZmBADH2a* and *Zm BADH2b*.	Maize u6	2-acetyl-1-pyrroline	Directed mutagenesis	*Agrobacterium*-mediated transformation.	(Wang et al., 2021)

## Metabolite engineering using CRISPR/Cas9 in plants

**Figure 2:**
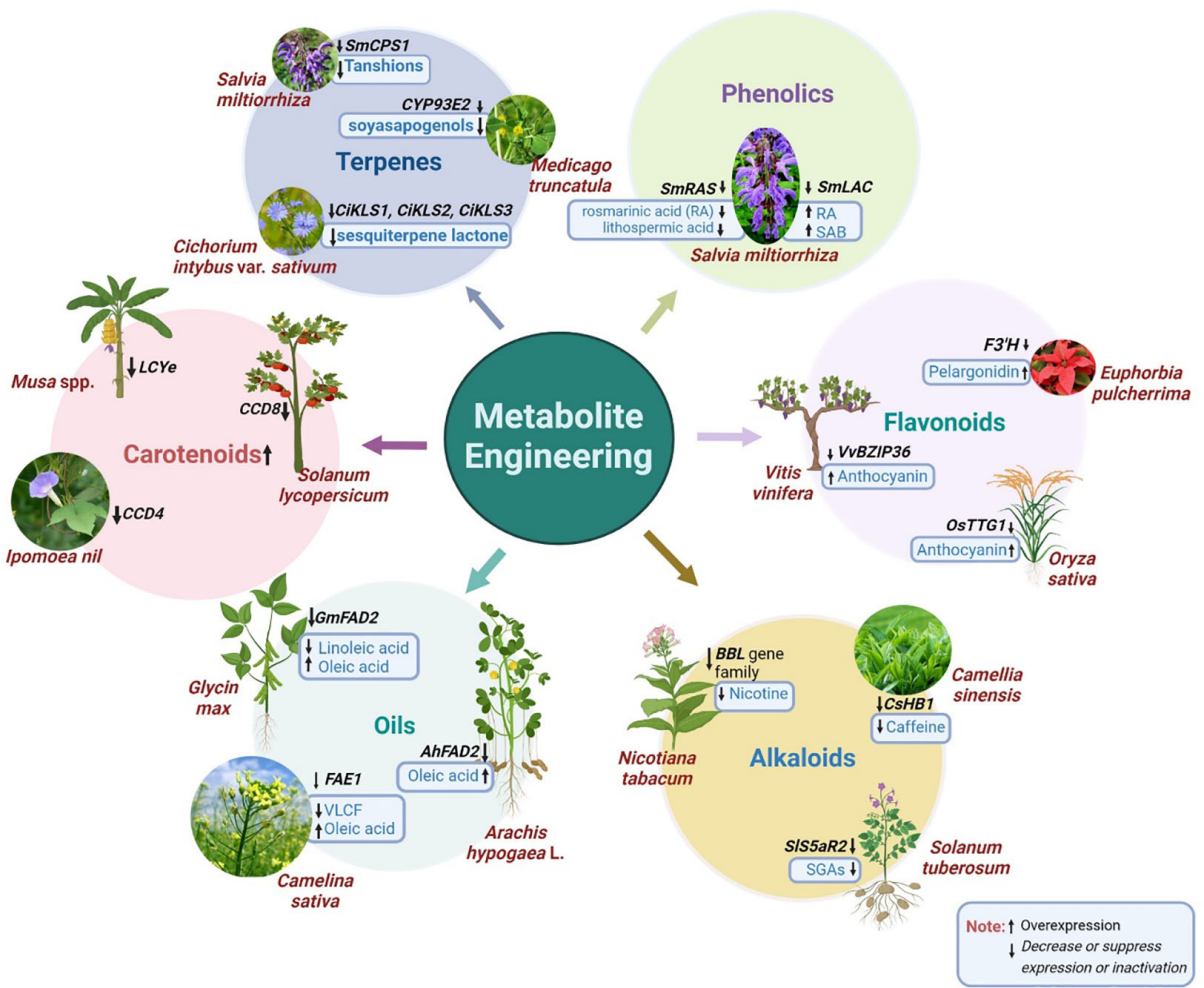
Schematic representation of metabolite engineering in plants.

Production of specific secondary metabolites can be increased/decreased by CRISPR/Cas technology. A pictorial representation of the experimentally proved CRISPR-based metabolite engineering of selected genes and their resulting changes in the metabolites is presented in **Figure 2**.

### Carotenoids

Carotenoids are tetraterpenoids which are natural pigments responsible for colours mainly in fruits and vegetables. In photosynthetic species, carotenoids play several roles such as in photosynthesis, phytohormone production, photoprotection, and pigmentation (Sun et al., 2022). They are secondary metabolites derived from the isoprenoid pathway. They are lipophilic and usually accumulate in the organs of plants. CRISPR/Cas-mediated genome editing has been reported for enhancing carotenoid accumulation in many plants. This approach has been reported to improve the accumulation of *β*-carotene in rice endosperm by editing the *OsOr* gene (Endo et al., 2019). A relatively large DNA fragment of 5.2 kb carotenoid biosynthesis cassette was inserted using an optimized CRISPR/Cas9-based method generating marker-free high carotenoid-enriched rice (Dong et al., 2020). This approach has also developed a *β*-carotene-enriched banana with the accumulation of *β*-carotene content up to 6-fold (~24 μg/g) by targeting the fifth exon of the lycopene epsilon-cyclase (*LCY*ϵ) (Kaur et al., 2020). Two key genes *Psy1* and *CrtR-b2* of the carotenoid pathway were edited by CRISPR/Cas9 resulting in a change in colour and the fruits. Carotenoid accumulation performs a key role in the colour shift (D’Ambrosio et al., 2018).

Strigolactones (SLs) are plant secondary metabolites that are derived from carotenoids. Carotenoid cleavage dioxygenase (*CCD*) is responsible for the regulation of carotenoid accumulation in plants. CRISPR/Cas targeted editing of carotenoid cleavage dioxygenase7 (*CCD7*) helps in fine-tuning strigolactone biosynthesis that is responsible for the change in plant architecture traits in rice (*Oryza sativa*) (Butt et al., 2018). In tomato (*Solanum lycopersicum*), CRISPR/Cas9 was used to precisely knock out the *CCD8* gene that helps in a significant reduction of orobanchol (SL) content thereby increasing carotenoids level by increasing the expression of genes related to carotenoid biosynthesis (Bari et al., 2019). In white flowered *Ipomoea nil*, successful knockout of *lnCCD4* using the CRISPR/Cas system resulted in producing pale yellow petals with an increase in total carotenoid content up to 20-folds (Watanabe et al., 2018). Mutation-mediated by CRISPR/Cas9 system at the *DFR-B* gene which is involved in anthocyanin biosynthesis generates several white flower mutants in *Ipomoea nil*. Thus, CRISPR/Cas9 technology enables to study various gene functions pertaining to colour changes in horticulture plants (Watanabe et al., 2017).

pCRISPR system was used to enhance both carotenoids and flavonoids by multi-target editing of related genes for carotenoid synthesis and other metabolic pathways in plants. In a study, genes related to carotenoid metabolism were targeted for enhancing lycopene accumulation. Using multiplex CRISPR/Cas9 genome editing system, five genes such as *SGR1*, lycopene ϵ-cyclase (*LCY-E*), beta-lycopene cyclase (*Blc*), lycopene β-cyclase 1 (*LCY-B1*) and lycopene β-cyclase 2 (*LCY-B2*) have been edited using *Agrobacterium*-mediated transformation. The site-specific genome editing efficiently edited the multiple genes thereby increasing the lycopene content by about 5.1-folds in the tomato fruit (Li et al., 2018). Tomato lycopene has also been reported to accumulate in mutagenic lines of *SlPGR1* generated through the CRISPR/Cas9 system (Ma et al., 2022a). In another study, knockout of *SlSGR1 in Solanum lycopersicum* had significantly increased chlorophyll and carotenoid level in comparison with that of the wild type. In those mutants, lycopene content was also increased suggesting that various genes (nine DEGs: *Fab G, AP2a, DDTFR8, RIN, LOXB, ERF-D2, LOXC, ACO1*, and *ACO6*) were affected by the knockout of *SlSGR1*. The study could further help in elucidating the role of *SRG1* gene involved in chlorophyll and carotenoid biosynthesis pathway (Kim et al., 2023).

### Phenolics compounds

Polyphenols comprise of phenolic acids, flavonoids, tannins, lignans, and coumarins (Ma et al., 2022b). Phenolics are the natural antioxidants and the most abundant group of phytochemicals in whole grains and are available in different forms: soluble-free, soluble-conjugated, insoluble-conjugated, and esterified forms. Among the phenolic acids in the whole grain, protocatechuic acid (Goufo et al., 2014), *p*-coumaric acid (Zhang et al., 2020a), ferulic acid (Acosta-Estrada et al., 2014), sinapic acid (Wu et al., 2009), and vanillic acid (Tanaka et al., 2019) were the main rice phenolics (Zaupa et al., 2015). In the Chinese traditional medicinal plant *Salvia miltiorrhiza*, the rosmarinic acid synthase gene (*SmRAS*) in the phenolic acid biosynthesis was edited using CRISPR/Cas9 system. In the successfully edited homozygous mutants, expression of *RAS* was reduced and there was a decrease in the content of the phenolic acids including rosmarinic acid (RA) and lithospermic acid. In addition, the level of rosmarinic acid’ precursors, particularly 3,4-dihydroxyphenyllactic acid was increased due to the *RAS* mutation. This finding indicated that CRISPR/Cas9 is an effective system to identify important genes related to biosynthetic pathway (Zhou et al., 2018). In another study, a negative regulator bZIP2 in phenolic acid biosynthesis had been targeted for mutagenesis in *Salvia miltiorrhiza* using CRISPR/Cas9 approach leading to the increase in phenolic acid content (Shi et al., 2021). This gives a potential method to manipulate metabolic networks for the quality improvement of medicinal herbs.

Laccases belong to the multicopper oxidase family which is involved in the biosynthesis pathway of phenolic compounds such as salvianolic acid B (*SAB*) in *Salvia miltiorrhiza.* Multiple genes of the laccase family (*SmLAC*) were knocked out using the multiplexed CRISPR/Cas9 system targeting the conserved domains. Expression of the laccase genes and the biosynthetic key genes of phenolic acid was greatly reduced. In the gene-edited lines, *RA* and *SAB* accumulation was decreased while the lignin content was nearly undetectable suggesting the key role of *SmLAC* genes in development, lignin formation, and phenolic acid biosynthesis (Zhou et al., 2021).

### Flavonoids

Flavonoids are phenolic secondary metabolites produced by plants as a defense mechanism. They are also responsible for the development of the colour and aroma in fruits and flowers. They are well known for their high antioxidant, anti-inflammatory, and anti-carcinogenic properties. Flavonoids are classified as flavones, flavonols, flavanones, flavanols, and anthocyanins (Wang et al., 2018). Among them, anthocyanin is the main flavonoid in pigmented rice varieties (Chen et al., 2019). Studies on the anthocyanin biosynthesis pathway have been carried out using CRISPR/Cas system and gene-specific mutations were induced with high efficiency in rice. CRISPR/Cas9 technology successfully edited *OsF3’H, OsDFR, OsLDOX* (Jung et al., 2019), and *OsTTG1* (Yang et al., 2021) with high efficiency in anthocyanin biosynthesis. Fruit colour in tomatoes is determined by the accumulation of carotenoids, flavonoids, and the degradation of chlorophyll. Multiplex gene editing of three genes related to fruit colour namely *PSY1*, *MYB12*, and *SRG1* was achieved using the CRISPR/Cas9 system. Using this approach, red coloured fruit cultivar was engineered to obtain different coloured fruits including yellow, brown, pink, light yellow, pink-brown, yellow-green, and light green (Yang et al., 2023). Phloridzin, the major dihydrochalcones (DHCs) present in the leaves of cultivated apples, and its precursor phloretin are important in the treatment of degenerative diseases such as Alzheimer’s disease (Viet et al., 2013). CRISPR/Cas9 induced *MdPGT1* edited lines exhibit reduced foliar phloridzin content to a comparable level with normal growth while 76.7-78.4% reduction was obtained in those knockdown lines with growth impairment. The study highlighted the contributions of genes involved in phloridzin biosynthesis and also those phytohormones involved in the effects of growth due to a reduction in phloridzin (Miranda et al., 2023).

CRISPR/Cas 9 system has been an efficient tool for increasing the flavonoids content of Tartary buckwheat (*Fagopyrum tataricum*) by precise editing of the *FtMYB45*, a transcription factor that negatively regulates the flavonoid biosynthesis (Wen et al., 2022). In soybeans (*Glycine max*), CRISPR/Cas9 mediated targeted mutagenesis of *GmUGT* in flavonoid biosynthesis enhanced the resistance against leaf-chewing insects (Zhang et al., 2022b). Knockout of *F3’H* by CRISPR/Cas9 system in red flowering poinsettia (*Euphorbia pulcherrima*) cultivar resulted in the accumulation of pelargonidin (Nitarska et al., 2021). In grapevine (*Vitis vinifera*), *VvBZIP36* acts as a negative regulator of anthocyanin biosynthesis. Knocking out of the gene by CRISPR/Cas9 approach resulted in the accumulation of anthocyanin metabolites (Tu et al., 2022). Isoflavonoids are derived from the phenylpropanoid pathway and include a variety of secondary metabolites. Multiplex CRISPR/Cas9 system has targeted *GmF3H1, GmF3H2*, and *GmFNS-1* which are the key enzymes for isoflavonoid biosynthesis in soybean [*Glycine max* (L.) Merr.]. The targeted mutagenesis helps in increasing the isoflavone content in soybean seeds and resistance to soybean mosaic virus (SMV) (Zhang et al., 2020b).

### Alkaloids

Opium poppy (*Papaver somniferum* L.) contains important secondary metabolites called benzyl isoquinoline alkaloids (BIAs) (Jablonická et al., 2016). BIAs are important in their application in the pharmaceutical market and research (Beaudoin and Facchini, 2014). BIAs such as morphine and thebaine were drastically reduced in genome-edited plant lines after the *4’OMT* and *4’OMT2* gene were knocked out using the CRISPR/Cas9 technology. The study gives an idea for application of CRISPR/Cas 9 in *Papaver* species in future studies for BIAs metabolism and its biosynthesis (Alagoz et al., 2016). Enzymes of the berberine bridge enzyme-like (BBL) family were involved in the final step of tobacco alkaloid formation. In order to get nicotine-free non-transgenic *Nicotiana tabacum*, CRISPR/Cas editing approach was applied to knock out the Berberine Bridge Like *(BBL)* gene family (Schachtsiek and Stehle, 2019).

Caffeine is an important purine alkaloid present in tea that accounts for more than 90% of the total alkaloids. Many transcription factors (TFs) such as basic helix-loop-helix (bHLH), WRKY, GRAS [(acronym derived from three proteins gibberellic-acid insensitive (GAI), repressor of GAI (RGA) and scarecrow (SCR), myeloblastosis (MYB), and NAC (acronym derived from three TFs: no apical meristem or NAM), *Arabidopsis thaliana* activation factor (ATAF1-2) and cup-shaped cotyledon (CUC2)] account for the regulation of purine alkaloid biosynthesis (Zhu et al., 2019). Another transcription factor family homodomain-leucine zipper (HD-Zip) also plays an important regulatory role in the secondary metabolite synthesis in tobacco, rice, and other plants (Sun et al., 2020). Transcription factor gene *CsHB1* belonging to the HD-Zip family regulates the caffeine biosynthesis gene *yhNMT1*. Using CRISPR/Cas approaches, *CsHB1* was mutated directionally resulting in the decrease of the expression of *CsHB1* and *yhNMT1* by 65% and 93% respectively. The lower expression of the two genes causes a decrease in caffeine accumulation by 97.5% in tea callus (Ma et al., 2021).

Steroidal glycoalkaloids (SGAs) are toxic and play a major role in defence against a wide range of pathogens and predators (Friedman, 2002, Friedman, 2006). In tomatoes, SGAs like α-tomatine and dehydrotomatine are accumulated in mature green fruits, leaves and flowers. The gene *SlS*5*α*R2 which is responsible for the C5α reduction in α-tomatine biosynthesis was knocked out by CRISPR/Cas9 mediated genome editing resulting in the significant decrease of the α-tomatine level and accumulation of dehydrotomatine (Akiyama et al., 2019). SGAs are present in every part of the cultivated potato (*Solanum tuberosum*). Due to the high content of SGAs, the use of the above-ground part of the potato is limited. Zheng et al. (2021) knocked out the *StSSR2* gene from potato plants using CRISPR/Cas9 system. The mutant lineage was found to produce a decreased level of SGAs in both leaves and tubers. *Symphytum officinale*, popularly known as comfrey, is a medicinal plant with analgesic, anti-inflammatory, and proliferative properties. It also contains pyrrolizidine alkaloids in its tissues, which are toxic besides their pharmaceutical properties. CRISPR/Cas9 gene editing was used to edit the *HSS* gene encoding homospermidine synthase (HSS), the first enzyme involved in PA biosynthesis. HSS catalyzes the formation of homospermidine as the exclusive substrate for PAs. Reduced levels of homospermidine were obtained in single *hss* allele edited mutants which in turn reduced PA levels; whereas no PA was detectable in mutants that have mutations in both the alleles (Zakaria et al., 2021).

### Oils

Oilseeds are a great source of potent bioactive compounds such as flavonoids, polyunsaturated fatty acids (PUFA), organosulphur compounds, phenolic compounds, phytosterols) that have the potential for pharmaceutical, agricultural and cosmetic industries (Morya et al., 2022). Important oil seed crop *Camelina sativa* had high PUFA. Most part of PUFAs is linolenic acid (30-40%) that is susceptible to oxidation (Fröhlich and Rice, 2005). The quality of the oil depends on the composition of the fatty acids as high PUFA linolenic acid had a shorter shelf-life due to rancidity. Higher content of monounsaturated fatty acids (MUFA) such as oleic acid in the oil is preferable for its higher oxidative stability and nutraceutical benefits. CRISPR/Cas9 system has been employed to knock out fatty acid desaturase 2 (*FAD2*) to reduce the PUFA while increasing the oxidatively stable oleic acid (Jiang et al., 2017; Morineau et al., 2017). Three *FAD2* genes were targeted in hexaploid *Camelina* for mutagenesis by the CRISPR/Cas9 system which yielded three different combinations of single, double, and triple mutants with various lipid profiles with the accumulation of 10 to 60% oleic acid (Morineau et al., 2017). In another study in *Camelina sativa*, mutation in *FAD2* by the CRISPR/Cas editing system changed the fatty acid profile of the seed oil, particularly an increase of over 50% oleic acid in the fatty acid composition along with a decrease in linoleic acid synthesis (from 16% to less than 4%) and linolenic acid (35% to less than 10%) (Jiang et al., 2017). In yet another study in *Camelina sativa*, three fatty acid elongase 1 (*FAE*1) alleles were targeted using CRISPR tools which resulted in a change in the fatty acid composition of the seed. The knockout mutants had fatty acids with increased levels of oleic acid or linolenic acid but the level of very long-chain fatty acids (VLCFs) was significantly reduced (Ozseyhan et al., 2018).

In the oil-producing allotetraploid crop peanut (*Arachis hypogaea* L.), CRISPR/Cas approach had precisely modified *AhFAD2* genes for increasing oleic acid content that might be useful for peanut breeding programs (Yuan et al., 2019). CRISPR/Cas9 editing technology was used in the legume crop soybean (*Glycine max*) to mutate the microsomal omega-6 desaturase gene *FAD2-2*. The transgenic lines recorded increased oleic acid (up to 65.58%) and decreased linoleic acid (16.08%). Soybean fatty acid desaturase encoding genes *GmFAD2-1A* and *GmFAD2-1B* were knocked out using the CRISPR/Cas9-mediated gene-editing technology. Accumulation of oleic acid increased from 11% to 40-50% in the *fad2-1a* and *fad2-1b* single mutants while *fad2-1a/fad2-1b* double mutants reached 85%. Linoleic acid reduction was observed from 57% to 2% in the *fad2-1a/fad2-1b* double mutants (Fu et al., 2022). Enhancing monounsaturated fatty acids (MUFA) while decreasing the PUFA content using CRISPR/Cas system is an important achievement for oilseed improvement. CRISPR/Cas9 multiplex genome editing had been used to achieve high oleic oil palm by targeting the fatty acid desaturase 2 *(FAD2)* and palmitoyl-acyl carrier protein thioesterase *(PAT)* genes. The two genes are associated with fatty acid metabolism in oil palms. Mutants with single- and double-knockout of both genes were generated which leads to oleic acid accumulation in oil palm (*Elaeis guineensis*) (Bahariah et al., 2023).

### Terpenes

Saponins are large groups of triterpene or steroid glycosides in the *Medicago* genus; haemolytic sapogenins in particular exhibit biological and pharmacological activities while soyasapogenol saponins lack those properties (D’Addabbo et al., 2020; Maestrini et al., 2020). *CYP93E2* and *CYP72A61* genes are responsible for the biosynthesis of soyasapogenol B, the most abundant soyasapogenol in *Medicago* spp. With the application of CRISPR/Cas technology in *Medicago truncatula*, the knock-out *CYP93E2* mutant did not produce soyasapogenols and was diverted for the production of valuable hemolytic sapogenins (Confalonieri et al., 2021). Chicory (*Cichorium intybus* var. *sativum)* is an important plant for the production of fructose polymer inulin, a low-calorie sweetener and prebiotic. During inulin extraction, bitter-tasting compounds sesquiterpene lactones (STLs) need to be removed. Germacrene A synthase (CiGAS) is the first enzyme for the dedicated step in STL synthesis. CRISPR/Cas9 system was used to edit the four genes that encode the CiGAS enzyme which showed complete elimination of STL synthesis (Cankar et al., 2021).

*Salvia miltiorrhiza* is a highly prized Chinese traditional medicinal herb due to the presence of the diterpene compounds, tanshinones that include cryptotanshinone, tanshinone IIA and tanshinone I along with the phenolic acids including rosmarinic acid, salvianolic acid, and lithospermic acid. The committed diterpene synthase gene (*SmCPS1*) involved in tanshinone biosynthesis was precisely knocked out using CRISPR/Cas9. In the homozygous mutants, the tanshionones, especially cryptotanshinone, tanshinone IIA, and tanshinone I were completely missing without affecting the phenolic metabolites while in chimeric mutants tanshionones were still detectable. CRISPR/Cas9 editing tool appears as a promising system to study the pathway elucidating secondary metabolites (Luo et al., 2014).

### Aroma

Plants accumulate a variety of fragrance-producing compounds in special cells, glands, or ducts located in different aerial and underground parts. The fragrance-producing compounds are low in volume but highly concentrated, and volatile when exposed to air at ordinary temperatures (Chakraborty et al., 2022). The aromatic compounds are valued for their usage in perfumery, soaps, cosmetics, pharmaceuticals, confectionery, and other related human benefits. Aroma is one of the most important grain quality characteristics in rice. Rice is broadly classified as aromatic and non-aromatic based on the presence of fragrance. Among over 200 volatile compounds identified in fragrant rice, 2-acetyl-1-pyrroline (*2-AP*) is the key component responsible for fragrance production in rice. The gene encoding betaine aldehyde dehydrogenase (*BADH2)* on chromosome 8 is the key gene for controlling the fragrance in rice. Shao et al. (2017) edited the fragrance gene *BADH2* in Zhonghua 11 which resulted in an additional T base at the first exon of *BADH2*. The expression of the *BADH2* was reduced in the mutant with the increase of 2-acetyl-1-pyrroline content as compared to the wild type (Shao et al., 2017). Tang et al. (2021) implemented CRISPR/Cas9 to target the intron 2 splice donor site (GT) of the *OsBADH2* gene. As a result of the changes at the gene’s splicing site, exon2 was not spliced out due to the change in the reading frame leading to premature termination codon in exon 3. The loss of *OsBADH2* function increases the 2-AP accumulation in the mutants (0.08 mg/Kg) while *2-AP* was not detected in the wild type (Tang et al., 2021). CRISPR/Cas system had successfully created novel allelic variations of *OsBADH2* with mutations located within -17bp to +15bp of the sgRNA flanking regions in the 7^th^ exon of *OsBADH2*. The allelic variation contributed to the aroma in the elite, non-aromatic rice variety ASD16 (Ashok Kumar et al., 2020). Hui et al. (2022) created new alleles of *OsBADH2* using CRISPR/Cas9 gene editing technology with the genetic background of the japonica Ningjing1 (NJ1), indica Huang Huazhan (HHZ). New alleles of *BADH2* (nj1-cr^BADH2^-1, nj1-cr^BADH2^-2, hhz-cr^BADH2^-1, hhz-cr^BADH2^-2) had accumulated more 2-AP with moderate fragrance compared with the wild type. Further, a three-line hybrid variety B-Tao-You-Xiangzhan (BTYXZ) with increased aroma had resulted from the cross between the HHZ background new alleles with the CM lines Taonong 1A (TNA1). CRISPR/Cas9 technology efficiently edited the *BADH2* genes to produce aroma in non-aromatic *japonica* and *indica* rice varieties (Hui et al., 2022).

Genetic mutations in *BADH2* in other plants also help in the production of 2-AP. In maize, no equivalent aromatic germplasm had been described earlier. With the use of the CRISPR/Cas9 technology, the world’s first aromatic maize was created by simultaneous genome editing of the two maize *BADH2* homologs, *ZmBADH2a* and *ZmBADH2b.* Double mutants *zmbadh2a-zmbadh2b* were noticeable with popcorn scent but not in single (*zmbadh2a* and *zmbadh2a*) or wild mutants. Fresh kernels were detected with 0.028 mg/kg *2-AP* while the dried mature seeds with 0.723 mg/kg 2-AP in the case of the double mutants (Wang et al., 2021). In another study, fragrant sorghum (*Sorghum bicolor* (L.) Moench) lines have been generated with high accumulation of 2-AP in roots and leaves through CRISPR/Cas9-mediated knockout of *SbBADH2* (Zhang et al., 2022a).

## Gene editing challenges

In most genome editing tools, off-target binding is a common technological challenge. An off-target effect arises because of discrepancies in the ability of the repeat variable dinucleotide (RVD) to recognize and bind its target sites. If the off-target cleave is extensive, it can outrun the repairing capacity of DNA leading to the death of the modified cell or organisms (Carroll, 2011). CRISPR/Cas9 system is easily applicable in almost all living systems because of their low cost and easy-to-use tools. The only inherent problem with the use of the CRISPR/Cas9 system is the rise of a few non-specific double strand breaks (DSBs) in genome regions upstream of the protospacer adjacent motif (PAM) (Jiang and Doudna, 2017). This may not be a concern with plants’ genome editing as most of the potential off-target sites have no report of anomalous phenotypes (D’Ambrosio et al., 2018). The frequency of off-target mutations by the CRISPR/Cas system is much lesser than those reported in the early phase of gene editing research involving chemical or physical mutagenesis or by ZFNs or TALENs. A probable mechanism to handle or leverage this problem could be the implementation of bioinformatics tools alongside gene editing tools. Whole genome sequences are currently available for many organisms, which makes it easier to design highly specific gRNAs, thereby enabling gene editing technologies for enhancing the secondary metabolite concentrations (Kim et al., 2017; Rees et al., 2017).

The generation of off-targets is also related to the expression levels of nuclease. Plasmid DNA transfection and other viral vectors though can deliver nuclease at high efficiency, but often they mediate high-level nuclease expression and for longer expression duration, which is a major cause leading to an increase in off-target mutations (Roos et al., 2007). Recent developments showed that virus-like particles which utilize the normal viral vector components without the viral genome could be a better choice as delivery tools. Nuclease encapsulated in such tools can be effectively delivered in the host cells without any loss in the nuclease activity. We briefly discussed the challenges that are encountered in the different systems below:

### Cell repair system

**Figure 3:**
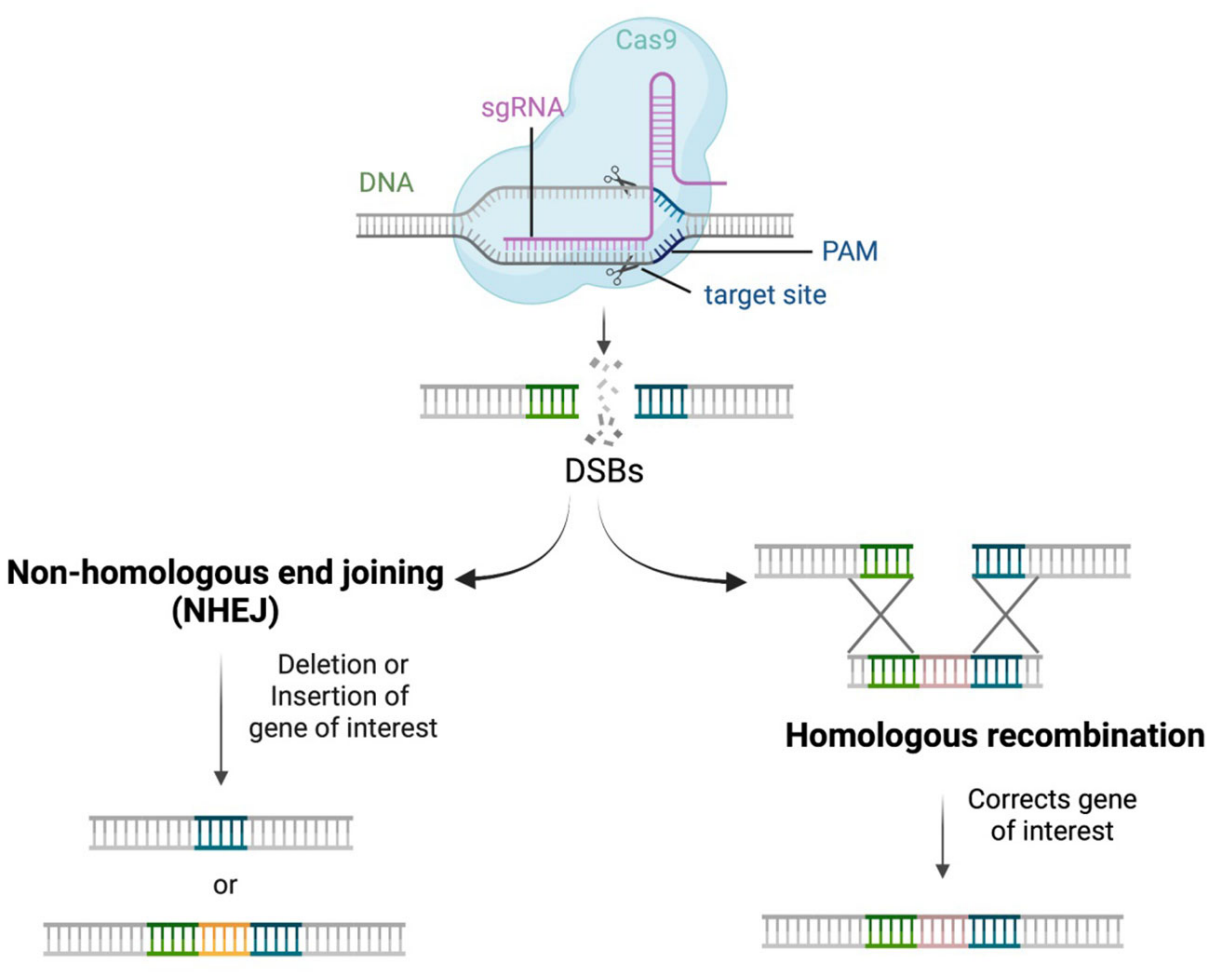
DNA repair mechanism mediated by CRISPR-Cas system.

Double stranded breaks (DSBs) are usually made by editing tools and the cell repair mechanism repairs these DSBs by either double-strand homologous recombination (HR) or non-homologous end joining (NHEJ). Homologous recombination usually happens when homologous DNA fragments are available resulting in gene replacement. NHEJ-mediated repair makes arbitrary changes in the nucleotides by either insertion or deletions (Indels) leading to precise alterations. Unlike the usual DNA-repair mechanism, CRISPR/Cas system can generate precise DSBs (**Figure 3**) that can be repaired by either of the above two mechanisms thereby making a desired gene alteration and finally altering the expression of the metabolic pathway (Yan et al., 2020). Gene arrangement, gene knockout, and gene replacement resulting from gene editing technologies are the main ways to engineer genes or genomes to enhance the secondary metabolite quantity of plants (Weeks et al., 2016). The arbitrary changes in the nucleotide may also cause frameshift mutation leading to unwanted knockouts of genes which is still a challenge.

### Promoter region

The expression of single guide RNA (sgRNA) depends on the type of promoter region for the function of RNA polymerase. In monocotyledons, the frequently used sgRNA promoters are *OsU6*, *Os6a*, *OsU6b*, and *OsU6c* while in dicotyledons, the suitable sgRNA promoters include *U6*, *AtU6-1*, and *AtU6-29* (Jiang et al., 2013). While in some plants like grapes and cotton, successful editing has been accomplished using endogenous U6 promoter (Long et al., 2018; Ren et al., 2021). Regulatory genes were targeted through CRISPR/Cas 9 approach for tissue-specific gene editing. Several studies have reported the use of tissue-specific promoters in the root (Li et al., 2021), leaves (Sanjari et al., 2019; McLellan et al., 2020), flowers (Sanjari et al., 2019), and seeds (Heang and Sassa, 2012). A dual promoter has been used in a tomato model system where Cas9/sgRNA expression was driven by *PPC2p* (fruit specific) and 35S *CaMV* (constitutive) promoters (Feder et al., 2020). Promoters regulate gene expression and the widely applicable promoter should not have any downstream transcriptional initiation sites.

### Polyploidy

Plants possessing polyploid genomes have large repetitive sequences, higher gene copy numbers, and duplication of the genes evolved with a different function. Such complexity of the polyploids is a challenge for gene editing to achieve desired mutations. Dosage effects are also prevalent among the paralogous copies of the genes. This is another challenge in gene editing, particularly in gene knockout. In some plants, it is necessary to edit all the multiple alleles of the target genes to meet the desired trait mutant which is quite a challenging task (Li et al., 2017; Schaart et al., 2021).

### Transformation efficiency

*Agrobacterium* and particle bombardment have been widely used for the generation of gene-edited plants by CRISPR/Cas system. This requires the time-consuming process of mutant line generation through tissue culture, accompanied by other challenges such as the lower frequency in the production of stable transgenics, and lesser precision in particle bombardment. As the Cas9 and sgRNA are expressed constitutively, the chances of integration of the CRISPR/Cas9 DNA in the unknown target sites is very high along with a high probability of off-target cleavages. Delivery by *in vitro* transcripts (IVTs) or ribonucleoprotein complexes (RNPs), complexes of CRISPR/Cas9 into regenerative cells through particle bombardment is a way to achieve transgene-free and reduce off-target effects (Liang et al., 2018). Selfing has also proved to produce transgene-free wheat lines to segregate out the CRISPR/Cas9 construct (Sánchez-León et al., 2018). Generally, any off-target mutations are segregated through genetic crosses. In plants, direct cell injection such as microinjection, lipofection, or electroporation is not suitable due to the presence of a cell wall. Protoplast transfection is an alternative solution, for instance, Cas9RNPs were successfully introduced into wall-less protoplasts followed by tissue regeneration in lettuce (Woo et al., 2015). Even though these methods are successful in getting transgenic-free and reduce off-target effects, they are quite laborious, costly, and feasible in fewer plants (Mao et al., 2019). The study should be carried out to achieve an easy and simple method for transformation that also reduces structural damage to the intact plant cell. Fossi et al. (2019) reported genome instability such as aneuploidy or change in chromosomal structure in the protoplast-regenerated plants.

### Off-target effect with Cas9

Off-target binding of the Cas9 causes cleavage in non-targeted sites and lead to unwanted chromosome arrangements such as deletions, inversions, and translocations (Brunet and Jasin, 2018; Yin et al., 2022). A high number of off-target events can induce genotoxicity which is harmful to plants (Blattner et al., 2020). Guide sequences like 20-nucleotides and the PAM sequence are the factors that curtail an effective Cas9 where the AM site is for Cas9 binding. In the case of the gRNA region, mismatches at the 5’ end are better tolerated than at the 3’ end suggesting that a 8-12 bps at the 3’ end is crucial for target recognition (Semenova et al., 2011; Wiedenheft et al., 2011). However, the effects can be the opposite where some mismatches in the 5’ end caused considerable effect greatly affecting Cas9 activity (Fu et al., 2013). The sequence of the guide RNA and the Cas9 structures can be a target for manipulation to reduce off-target cleavages. Expression of the gRNA and Cas9 is one of the potential strategies to reduce off-target events. Modified gRNA with truncated 3’ ends or the addition of two extra G nucleotides in the 5’ end also yield better on-target and off-target ratios but low efficiencies of on-target genome editing (Cho et al., 2014).

### Cas9 variants

CRISPR/Cas9 system with spCas9 (isolated from *Streptococcus pyogenesis*) is the widely used system because of its high efficiency in generating DSBs at GC-rich target sites. The PAM site NGG (N – any nucleotide, G – guanine) is the recognition site for the spCas9. Despite the efficiency, Cas9 has difficulty in restriction in AT-rich sequences and high off-target bindings. To overcome the problem of high off-target bindings, Cas9 variants were employed to reduce non-specific binding reducing off-target effects (Kleinstiver et al., 2016). Mutation of the *RuvC* domain of the Cas9 had converted into nickase which ultimately reduces off-target effects.

Modified Cas9 nucleases are also introduced to alter its gene expression thereby reducing off-target events. Cas9 nickase with a mutated RuvC nuclease domain cleaves the DNA strand complementary to gRNA while Cas9 nickase with a mutated HNH nuclease domain cleaves only the DNA strand interacting with gDNA (Nishimasu et al., 2014). Dead Cas9 (dCas9) or catalytically inactive Cas9 are recruited without cleaving the target sgRNA. dCas9 merged with FokI nuclease to develop fCas9 which showed 140-fold increased specificity in Cas9-derived modified cells (Guilinger et al., 2014). The use of “paired nickases” where two gRNAs and Cas9 nickases are used to generate adjacent offset nicks at the target site to improve specificity (Mali et al., 2013; Ran et al., 2013a; Ran et al., 2013b). Truncated gRNAs which have been shortened at the 5’ end showed decreased mutagenic effects at off-target sites while increasing sensitivity towards the single or double mismatches at the gDNA : DNA interface (Fu et al., 2014).

Several SpCas9 variants have been developed for reducing the off-side effects, *eSpCas9* (1.1) harbouring K848A/K1003A/R1060A mutations that have decreased affinity of Cas9 with non-target DNA strand (Slaymaker et al., 2016). The *spCas9-HF2* variant was designed to cleave the off-target sites (Kleinstiver et al., 2016). HypaCas9 which possesses mutants in the REC3 domain stringently traps the HNH domain at the conformational checkpoint in off-target sites (Chen et al., 2017). The high-fidelity Cas9 variants such as HeFSpCas9 and evoCas9 variants required a high level of specificity and were more efficient than SpCas9 in maintaining the on-target sites (Casini et al., 2018; Lee et al., 2018). Three high-fidelity SpCas9 variants such as eSpCas9(1.1), SpCas9-HF2, and HypaCas9 were engineered to serve as C to T base editors (Xu et al., 2019). Nme2Cas9, a Cas9 variant from *Neisseria meningitides*, had evolved into four variants eNme2-C, eNme2-C.NR, eNme2-T.1, and eNme2-T.2 enable robust precision genome editing with a single specified pyrimidine nucleotide at PAMs (Huang et al., 2023).

## Advances and developments in gene editing

The inefficiencies of homologous recombination-mediated repair were largely overcome by precise base editors that irreversibly convert one base into another in the target DNA and thus enabling its wide application in genome editing (Kim, 2018). Editing activities of high-fidelity Cas9 variants have also been enhanced by linking sgRNA to a self-cleaving ribozyme (Kim et al., 2017). Anzalone et al. (2019) reported a novel method of genome editing, where prime editing gRNA (pegRNA), a complex of longer gRNA and Cas9H840 nickase, fused to an engineered reverse transcriptase enzyme. Prime editing allows all possible nucleotide substitutions such as the conversion of the base to base, indels without the need for DSBs or donor templates and this provides a new platform for genome editing. Tissue-specific promoters have been reported for their effect on the target gene expression in tissue-specific genome editing (Ali et al., 2020; Lei et al., 2021). Several studies have reported the use of tissue-specific promoters such as root (Li et al., 2019), guard cells, green tissues of leaves (Wang et al., 2015), fruits (Agarwal et al., 2017) and seeds including endosperm (Zhu et al., 2018). For a successful tissue-specific genome editing using CRISPR/Cas9 system, the choice for the promoter and endonuclease activity of the sgRNA/Cas9 complex is crucial (Guo et al., 2018). Since the secondary metabolites are synthesised and accumulated at various parts of the plant, tissue-specific genome editing has been widely utilized in metabolic engineering.

In another approach, effector domains or activation domains (ADs) are commonly used with dCas9 DNA binding domains for activating the CRISPR/Cas systems. Transcriptional activators binding to a nuclease-null Cas9 protein (dCas9) enabled an efficient expression of the target gene becoming a transcriptional activator PTA, also known as CRISPR activator (CRISPRa) (Mali et al., 2013; Armando Casas-Mollano et al., 2020). In the second generation, multiplexed gene activation of up to 10 genes, using 10sgRNAs was achieved using the synergistic activation mediator system (SAMS) (Konermann et al., 2015). This approach will help in the expression of genes involved in secondary metabolite production in plants specially those metabolites which are found in very limited quantities.

Maji et al. (2019) described the first small-molecule inhibitors of Cas9, a cell-permeable and stable compound of less than 500 Da. The CRISPR inhibitor does not have any interference with the Cas9-sgRNA complex formation but blocks the binding of the complex to the DNA thereby blocking its ability to cleave the DNA strand. The Cas9 inhibitors could precisely switch off the editing process thereby not allowing the long-term stability of Cas9 that results in more off-target mutations. In another approach, RNA editing for a programmable A to I replacement (REPAIR) system, a catalytically inactive Cas13 (dCas13) is fused with adenosine deaminase acting on RNA type2 (ADAR2) to direct adenosine to inosine deaminase activity to transcripts. This system could edit full-length transcripts containing pathogenic mutations (Cox et al., 2017). REPAIR system is further engineered to create a variant with high specificity and this is a promising RNA-editing platform with wide applications in biotechnology.

## Conclusion

The emerging technologies for gene editing particularly the CRISPR/Cas-based systems, and the availability of many advanced sequencing platforms have revolutionized biological research. With the development of genetic engineering, enhancing or down-regulating certain traits or pathways from their natural origin or even introducing novel biological functions have been possible for the improvement of secondary metabolite production in medicinal plants thereby meeting the market demands. However, there is a need to address many challenges to achieve the full potential of advanced CRISPR technologies. Even though the advanced gene editing system has shown great promise and flexibility in selecting desired traits, off target binding becomes a serious concern leading to many unwanted and catastrophic results. There is a scope for improvement in enhancing the specificity of the Cas proteins in the CRISPR systems. In the future, overcoming the challenges will give new horizons in understanding, exploring, and the ability to alter desired traits for increasing the bioactive content in many medicinal plants.

## Author contributions

The authors confirm their contribution to the paper as follows: AM, KK, SD: concept and design, AM, KK: draft manuscript preparation PP, ML: manuscript revision SD: finalize the manuscript. All authors contributed to the article and approved the submitted version.
